# Effect of Variable Selection Strategy on the Performance of Prognostic Models When Using Multiple Imputation

**DOI:** 10.1161/CIRCOUTCOMES.119.005927

**Published:** 2019-11-13

**Authors:** Peter C. Austin, Douglas S. Lee, Dennis T. Ko, Ian R. White

**Affiliations:** 1 ICES, Toronto, ON, Canada (P.C.A., D.S.L., D.T.K.).; 2 Institute of Health Management, Policy and Evaluation, University of Toronto, ON, Canada (P.C.A., D.S.L., D.T.K.).; 3 Sunnybrook Research Institute, Toronto, ON, Canada (P.C.A., D.T.K.).; 4 Department of Medicine, University of Toronto, ON, Canada (D.S.L., D.T.K.).; 5 Peter Munk Cardiac Centre, University Health Network, Toronto, ON, Canada (D.S.L.).; 6 Medical Research Council Clinical Trials Unit, University College London, United Kingdom (I.R.W.).

**Keywords:** death, hospitalization, incidence, myocardial infarction, probability

## Abstract

**Background:**

Variable selection is an important issue when developing prognostic models. Missing data occur frequently in clinical research. Multiple imputation is increasingly used to address the presence of missing data in clinical research. The effect of different variable selection strategies with multiply imputed data on the external performance of derived prognostic models has not been well examined.

**Methods and Results:**

We used backward variable selection with 9 different ways to handle multiply imputed data in a derivation sample to develop logistic regression models for predicting death within 1 year of hospitalization with an acute myocardial infarction. We assessed the prognostic accuracy of each derived model in a temporally distinct validation sample. The derivation and validation samples consisted of 11 524 patients hospitalized between 1999 and 2001 and 7889 patients hospitalized between 2004 and 2005, respectively. We considered 41 candidate predictor variables. Missing data occurred frequently, with only 13% of patients in the derivation sample and 31% of patients in the validation sample having complete data. Regardless of the significance level for variable selection, the prognostic model developed using only the complete cases in the derivation sample had substantially worse performance in the validation sample than did the models for which variables were selected using the multiply imputed versions of the derivation sample. The other 8 approaches to handling multiply imputed data resulted in prognostic models with performance similar to one another.

**Conclusions:**

Ignoring missing data and using only subjects with complete data can result in the derivation of prognostic models with poor performance. Multiple imputation should be used to account for missing data when developing prognostic models.

Prognostic models are mathematical or statistical models that combine information on patient characteristics to produce predictions about future patient outcomes (eg, subsequent mortality or future incidence of heart disease).^1^ Prognostic models permit informed clinical decision-making. They permit effective risk stratification so that effective therapies and interventions are targeted at the patients most likely to benefit. Examples of prognostic models include the Framingham Risk Score for predicting cardiovascular disease,^2^ the GRACE score for predicting mortality following hospitalization for acute coronary syndromes,^3^ and the EFFECT-HF mortality risk score for predicting mortality in patients hospitalized with congestive heart failure.^4^

Selection of variables for inclusion in a prognostic model is an important issue. Clinical knowledge and expertise combined with the existing literature often provide investigators with a lengthy list of candidate predictor variables. To increase use of a prognostic model by clinicians and to reduce the data collection burden on future users, it is often necessary to develop a parsimonious prediction model that uses only a subset of the candidate predictor variables. Despite their limitations, variable selection methods such as backward variable elimination and forward variable selection are popular with applied analysts.^5^

The occurrence of missing data is an important issue when using clinical data. Missing data occur when some variables are only measured on a subset of the subjects. Rubin developed a framework for addressing missing data.^6^ The framework is easiest to describe for a single incomplete variable. Data are said to be missing completely at random if the probability that a given variable is missing for a specific subject is unrelated to the value of that variable or of any other variable. Data are said to be missing at random if the probability that a given variable is missing for a specific subject is unrelated to the value of that variable, conditional on the observed values of other variables. Finally, data are said to be missing not at random if the probability that a given variable is missing for a specific subject is related to the value of that variable itself, conditional on the observed values of other variables. Developing a prognostic model using only subjects with complete data (ie, excluding those subjects with any missing data) can have at least 2 possible adverse consequences. First, the estimated standard errors for the regression coefficients would be unnecessarily large, as information would be lost by excluding subjects with any missing data. Second, if the data were missing at random and not missing completely at random, then the estimated regression coefficients could be biased. To address the problems presented by missing data, Rubin developed multiple imputation, which entails the creation of M (M>1) copies of the original sample in which missing data have been filled-in using a model for the missing data.^6^ Each of the M imputed datasets is complete, in that missing data are not present. In each of the M imputed datasets, a conventional statistical analysis is conducted. Estimated regression coefficients and their standard errors can be combined using Rubin’s Rules, which account for both within- and between-imputation variability.

An important issue when developing prognostic models is the validation of their performance. Validation refers to assessing the performance of the prognostic model in samples other than the one used for model development or derivation. Justice described different types of model validation or transportability.^7^ A model is described as displaying geographic transportability if it performs well in geographic locations different from the one in which it was developed. A model is described as displaying temporal transportability if it performs well in time periods different from the one in which it was developed. Before their widespread adoption in clinical practice, it is important that prognostic models undergo validation.

Despite the frequency with which missing data occur in clinical research and the need to develop parsimonious prognostic models, there is paucity of information on how to conduct variable selection when using multiple imputation. The issue is not straightforward because the variables selected by a given variable selection procedure could differ across the different imputed datasets (eg, the variable denoting systolic blood pressure could be selected for inclusion in the first imputed dataset, but not in the second imputed dataset). Wood et al described 9 different methods to conduct variable selection when using multiple imputation and evaluated the performance of these methods (we briefly describe these methods in the following section). Wood et al used Monte Carlo simulations to assess the performance of the 9 different variable selection schemes. They evaluated the variable selection methods in terms of their ability to correctly select variables from the true model and to exclude variables not in the true model.^8^

While the ability to correctly identify variables in the true model is important, equally important is the ability to develop prognostic models that display good performance when validated in independent samples that were not used for model development. The objective of the current study is to evaluate the performance of different variable selection methods for use with multiply imputed data when the evaluation criterion is the prognostic accuracy of the derived models when applied to independent validation samples. The paper is structured as follows: First, we review previously described methods for variable selection when using multiply imputed data. Second, we describe a case study used to compare the prognostic ability of models developed using different variable selection methods. Third, we report the results of our analyses. Finally, we summarize our findings and place them in the context of the existing literature.

## Statistical Methods for Variable Selection When Using Multiple Imputation

Wood et al conducted a simulation study to examine the performance of different methods for variable selection using backwards variable elimination in multiply imputed data.^8^ We describe the variable-selection methods described in their paper, using their terminology when referring to each method, and briefly summarize them in the appendix. While Wood et al examined the use of backwards variable selection, similar approaches could be used with other variable selection methods (eg, forward variable selection or shrinkage-based methods such as the least absolute shrinkage and selection operator^1^).

### Variable Selection Using Complete Cases (Complete)

This approach restricts the analytic sample to those subjects with complete data on all candidate variables. Conventional variable selection methods (eg, backwards variable selection) are applied in the sample consisting of all subjects with complete data.

### Single Stochastic Imputation (Single)

This approach uses a single imputed dataset for variable selection. For instance, variable selection can be conducted using the first imputed dataset.

### Separate Imputations (S1, S2, and S3)

This approach is a modification of the previous approach. Variable selection is conducted separately in each of the M imputed datasets. The analyst notes the variables that were selected for inclusion in each of the M imputed datasets. Once this has been done, there are 3 different approaches to selecting the variable for inclusion in the final prediction model. Approach S1 selects those covariates that were selected in at least one of the M imputed datasets. Approach S2 selects those covariates that were selected in at least half of the M imputed datasets. Approach S3 selects those covariates that were selected in all of the M imputed datasets.

### Stacked Imputed Datasets With Weighted Regressions (W1, W2, and W3)

This approach entails stacking the M imputed datasets into 1 large dataset and then conducting variable selection in this single stacked dataset. To account for the multiple observations for each subject, weights are incorporated into the regression model when conducting variable selection. Wood et al proposed 3 different sets of weights that could be used: W1: *w*=1/M, in which each subject is weighted using the reciprocal of the number of imputed datasets; W2: *w*=(1−*f*)/M, where *f* denotes the proportion of missing data across all variables; W3: *w*_*j*_=(1−*f*_*j*_)/M, where *f*_*j*_ denotes the proportion of missing data for variable *X*_*j*_. Using the third approach, a different set of weights is used when assessing the statistical significance of a given candidate predictor variable.

### Application of Rubin’s Rules for Variable Selection (RR)

The final approach involves using Rubin’s Rules at each stage of variable selection to determine the statistical significance of each candidate predictor variable included in the regression model at a given step in variable selection. Thus, when using backward variable selection, the full model is fit in each of the M imputed datasets and the estimated regression coefficients and their standard errors are pooled using Rubin’s Rules. The variable with the largest *P* value is then excluded from the regression model, and the process is repeated until all retained variables meet a prespecified level of statistical significance (eg, *P*≤0.05).

### Estimation of Regression Coefficients for the Selected Variables

Once the variables have been selected using a given variable selection method, the associated regression coefficients can be estimated in each of the imputed datasets and the regression coefficients and their standard errors can be combined using Rubin’s Rules to produce the final model (this step is obviously superfluous in the last variable selection approach, which explicitly applied Rubin’s Rules when conducting variable selection).

## Methods

The use of data in this project was authorized under section 45 of Ontario’s Personal Health Information Protection Act, which does not require review by a Research Ethics Board. The first author had full access to all the data in the study and takes responsibility for its integrity and the data analysis. The data sets used for this study were held securely in a linked, de-identified form and analyzed at ICES. While data sharing agreements prohibit ICES from making the data set publicly available, access may be granted to those who meet prespecified criteria for confidential access, available at www.ices.on.ca/DAS.

### Data Sources

The EFFECT (Enhanced Feedback for Effective Cardiac Treatment) Study was designed to improve the quality of care for patients with cardiovascular disease in Ontario.^9^ During the first phase (referred to as the EFFECT Baseline sample), detailed clinical data were collected on patients hospitalized with acute myocardial infarction or congestive heart failure between April 1, 1999 and March 31, 2001 at 85 hospital corporations in Ontario, Canada, by retrospective chart review. During the second phase (referred to as the EFFECT follow-up sample), data were abstracted on patients hospitalized with these conditions between April 1, 2004 and March 31, 2005 at 81 Ontario hospital corporations. Data on patient demographics, vital signs, and physical examination at presentation, medical history, and results of laboratory tests were collected for these samples.

For the current study, we restricted our sample to patients hospitalized with acute myocardial infarction. Data were available on 11 524 and 7889 patients hospitalized with a diagnosis of acute myocardial infarction during the first and second phases of the study, respectively. For the current study, the outcome was a binary variable denoting whether the patient died within 1 year of hospital admission. Candidate predictor variables were those 41 binary and continuous variables listed in Table 1; no categorical variables had >2 levels. The mean/prevalence of each of the 41 covariates along with that of the binary outcome are reported in Table 1 for each of the 2 phases of the study, along with the percentage of subjects with missing data for each variable. The outcome variable was not subject to missing data as it was obtained by deterministic linkage to a population-based registry of the vital status of all residents of Ontario. In the derivation sample, 13% of subjects had complete data (and 87% of subjects were missing information on at least one variable), while in the validation sample 31% of subjects had complete data (and 69% of subjects were missing information on at least one variable).

**Table 1.**
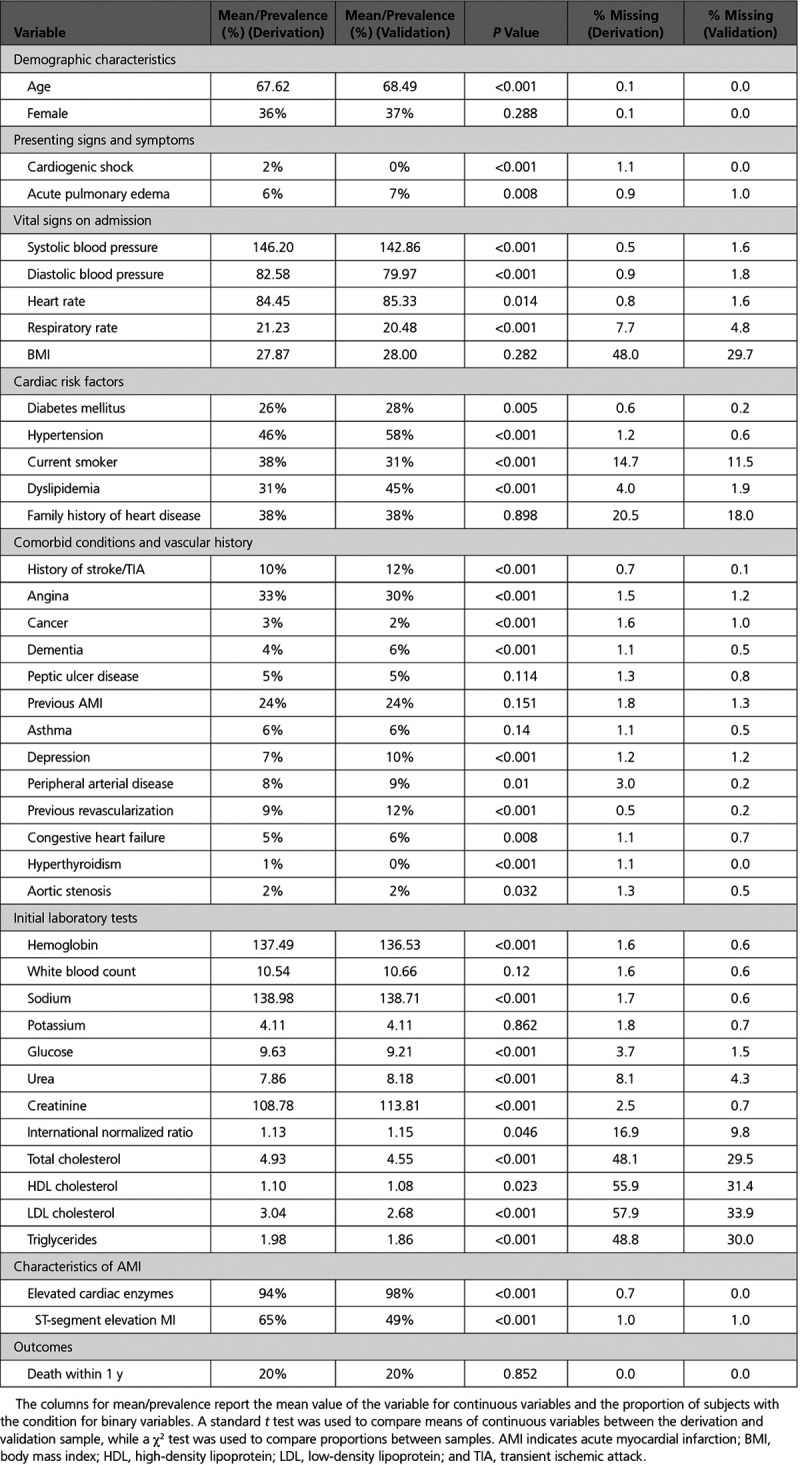
Description of Derivation and Validation Samples

In the derivation sample, 2310 (20.0%) subjects died within 1 year of hospital admission, while 1590 (20.2%) subjects in the validation sample died within 1 year of hospital admission. Given the use of 41 candidate predictor variables, the number of events per variable was 56 in the derivation sample and 39 in the validation sample.

### Statistical Methods

The EFFECT baseline sample was used as the derivation sample for model selection and estimation. The EFFECT follow-up sample was used as the validation sample for assessing the performance of the models estimated in the derivation sample.

Our aim was to examine ideal model performance in settings without missing data.^10^ As such, all of the imputation models included the outcome variable.

Multiple imputation was conducted separately in the derivation and validation samples. Imputation was conducted using a fully conditional specification approach using PROC MI in SAS (SAS/STAT version 14.1). Logistic regression models were used as the imputation models for the binary variables, while linear regression models were used as the imputation models for the continuous variables. All variables (including the binary outcome variable) were included in each imputation model (with the exception of the variable that was being imputed). No interactions or nonlinear terms were included. For each of the 2 samples, we set the number of imputed datasets (M) to be equal to the percentage of subjects with any missing data in the given sample.^11^ Thus, we created 87 imputed datasets for the derivation sample and 69 imputed datasets for the validation sample.

Variable selection was conducted using the 9 approaches described above (complete, single, S1, S2, S3, W1, W2, W3, and RR). For each approach, we considered 2 different significance levels for variable retention. First, backward variable selection was used with the criterion that the statistical significance of retained variables had to be ≤0.05. Second, backward variable selection was used with the criterion that the statistical significance of the retained variables had to be <0.157, which, for continuous or binary variables, is equivalent to the use of the Akaike Information Criterion.^1^ The second criterion was used as the first may be overly restrictive for developing prognostic models. Once the variables were selected using 1 of the 9 variable selection approaches, the coefficients for the final model were estimated in each of the 87 imputed versions of the derivation sample and the regression coefficients combined using Rubin’s Rules.

After a final regression model had been selected using each of the 9 approaches and its coefficients estimated, the estimated regression model was applied to each of the 69 imputed versions of the validation sample. The performance of the logistic regression model developed in the derivation sample was assessed in each of the 69 imputed versions of the validation sample. We used 4 different quantitative metrics for assessing the performance of the selected models: the c-statistic (equivalent to the area under the receiver operating characteristic curve), Nagelkerke’s generalized R^2^ statistic, the scaled Brier score, and the calibration slope.^1,12,13^ The Brier score is the mean squared prediction error (with smaller values of the Brier score denoting more accurate prediction). The scaled Brier score is defined as 
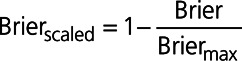
, where Brier_max_ denotes the maximum possible Brier score. The scaled Brier score ranges from 0% to 100%. In each imputed version of the validation sample, we regressed the observed binary outcome on the linear predictor computed using the regression coefficients estimated in the derivation sample. The calibration slope is the regression coefficient for the estimated linear predictor.

As our aim was to examine ideal model performance in settings without missing data, we evaluated the performance of the derived model in each of the imputed versions of the validation sample, rather than pooling predictions for each subject across the imputed datasets and evaluating performance based on these pooled predictions.^10,14^ For each of the 9 variable selection methods and for each of the 4 quantitative measures of model performance, there was no evidence that the distribution of the measure of model performance was non-normal across the imputed datasets (*P*>0.11 for the 36 applications of the Shapiro-Wilk test of normality when using *P*=0.05 for the variable selection criterion and when using *P*=0.157 for the variable selection criterion). Thus, we applied Rubin’s Rules and computed the mean of the estimates of model performance (eg, the c-statistic) across the 69 imputed versions of the validation sample.

Loess-based graphical methods were used to assess the calibration of each derived model when applied to the imputed versions of the validation sample.^15^ Each model developed in the derivation sample (and whose coefficients were subsequently estimated using Rubin’s Rules) was applied to each of the 69 imputed versions of the validation sample. A predicted probability of the outcome was obtained for each subject in each of these 69 samples. Loess-based graphical methods were used to assess the calibration of the derived model when applied to each of the imputed versions of the validation sample. The resultant 69 calibration curves were then averaged to obtain a final calibration curve.

In clinical practice, simple mean imputation may be used rather than multiple imputation. We therefore also compared model performance in this context. A single imputed version of the validation was created in which missing continuous variables were imputed using the mean of the observed values for that variable, while missing binary variables were imputed using the mode of the observed values for that variable. For each of the variable selection approaches, the regression model selected and estimated in the derivation sample (using the 87 imputed versions of the derivation sample) was applied to this single imputed version of the validation sample. The performance of the fitted model in this single validation sample was assessed using the c-statistic, the generalized R^2^ statistic, and the scaled Brier score.

## Results

Results are reported separately for the derivation and validation samples. A standard *t* test was used to compare the means of continuous variables between the derivation and validation samples while a χ^2^ test was used to compare the distribution of binary variables between the derivation and validation samples. In the derivation sample, the percentage of subjects with missing data for a given variable ranged from 0% to 57.9%, with a median of 1.4% (25th and 75th percentiles: 0.9% and 4.0%). In the validation sample, the percentage of subjects with missing data for a given variable ranged from 0% to 33.9%, with a median of 0.9% (25th and 75th percentiles: 0.5% and 1.9%).

### Variable Selection Using *P*=0.05 for Variable Retention

The variables selected using each of the variable selection approaches when a significance level of 0.05 was used for variable selection is reported in Table 2. Six variables were not selected using any of the variable selection approaches, while 9 variables were selected using all 9 variable selection approaches. The numbers of selected variables for the different variable selection methods were: 10 (complete case selection), 21 (S3), 27 (single sample selection and W3), 28 (W2 and Rubin’s Rules), 29 (W1), 31 (S2), and 36 (S1) (see first row of Table 2). The estimated odds ratios and associated 95% CIs for the predictor variables in each model are reported in Table 3. The regression coefficients for these models were estimated in each of the imputed versions of the derivation sample and were then pooled using Rubin’s Rules. Note that several of the estimated effects are not statistically significant. This is due to the final estimates and CIs for all models being estimated in all imputed datasets and then being pooled using Rubin’s Rules. By definition, all of the variables in the model whose variables were selected using applications of Rubin’s Rules were statistically significant (*P*≤0.05). Similarly, the use of the S3 and W3 algorithms resulted in all included variables being statistically significant. However, the following variable selection methods resulted in the inclusion of variables that were not significant after estimation using Rubin’s Rules: single (2 variables), S1 (9 variables), S2 (4 variables), W1 (2 variables), and W2 (2 variables).

**Table 2.**
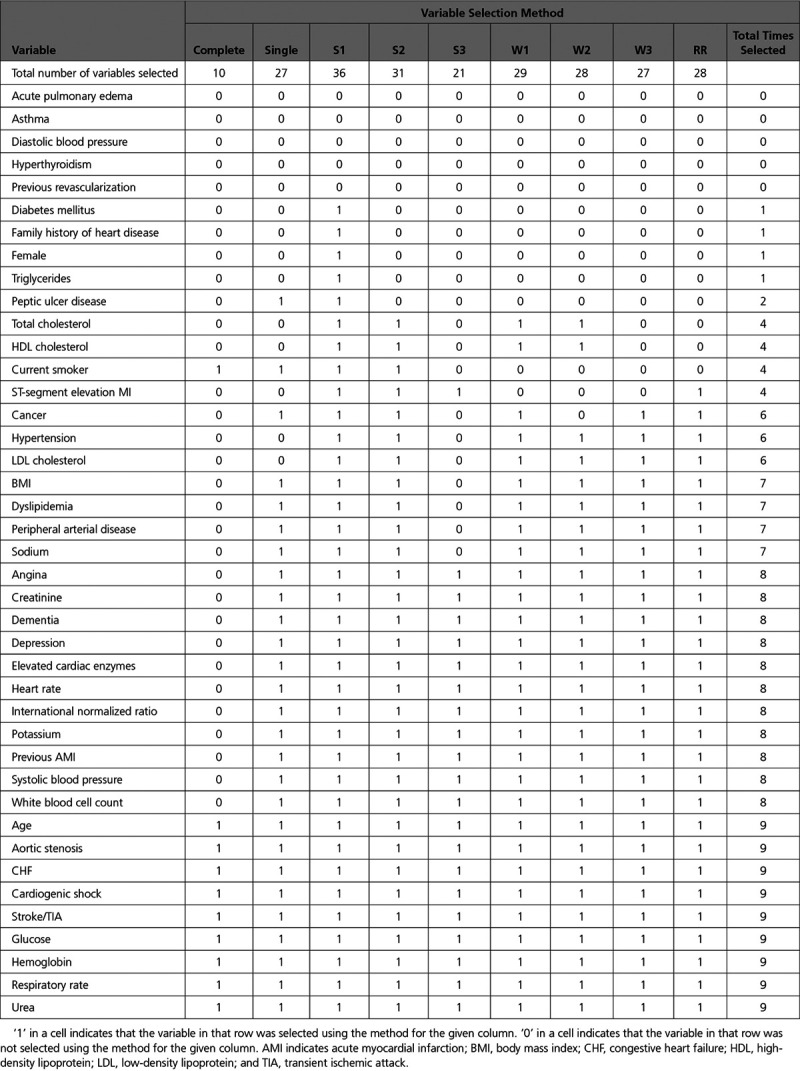
Variables Selected Using Each Variable Selection Approach When Using a 0.05 Significance Level for Variable Retention

The regression models reported in Table 3 (derived and estimated in the derivation sample) were then applied to each of the imputed versions of the validation sample. The mean of the model performance metrics (c-statistic, generalized R^2^ statistic, scaled Brier score, and calibration slope) across the 69 imputed versions of the validation sample are reported in Table 4. The most notable observation is that the model whose variables were selected using the complete cases had noticeable worse performance than did the other models across all 3 measures of model performance.

**Table 3.**
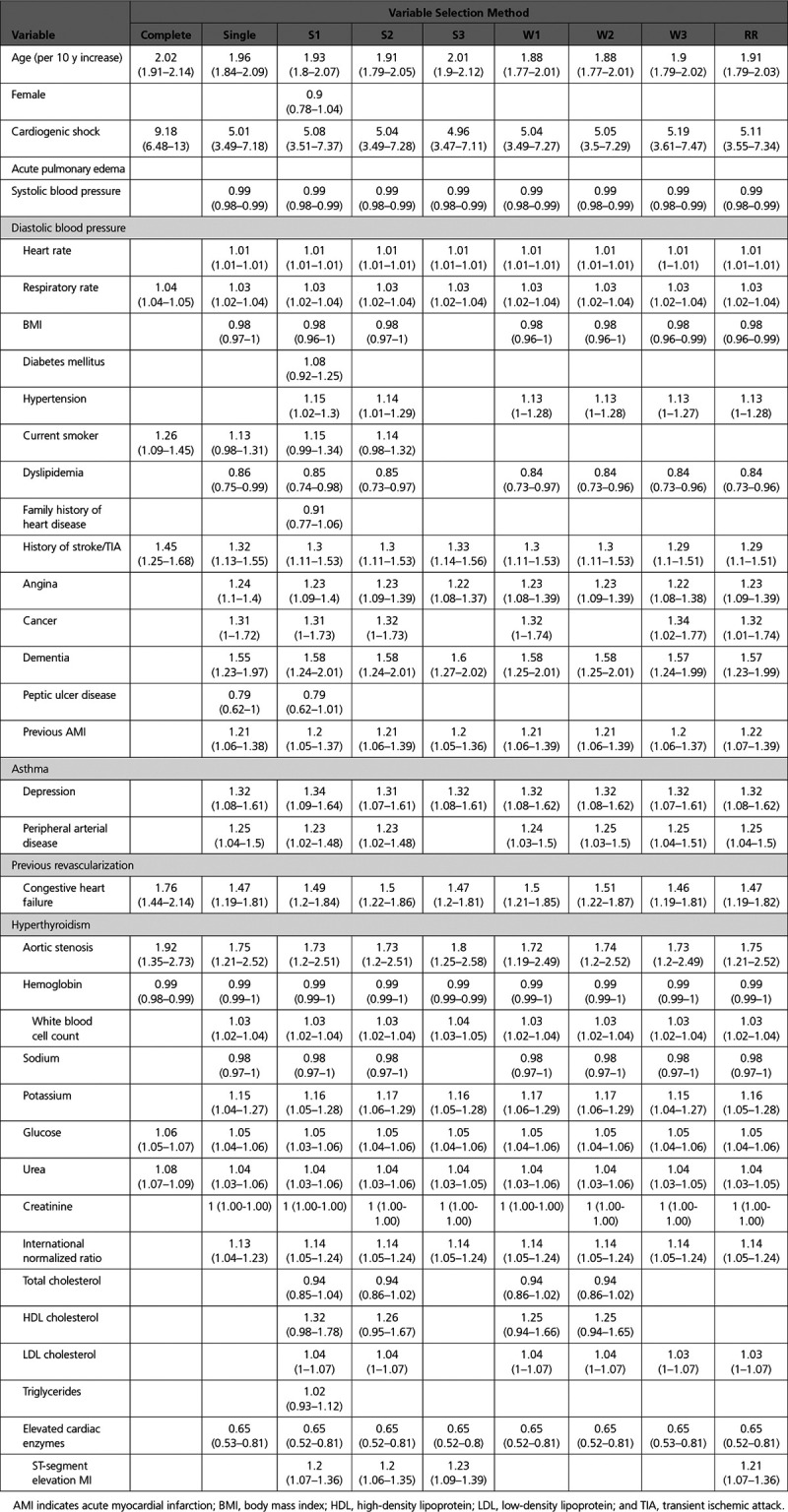
Estimated Odds Ratios and 95% CIs in the Derivation Sample When Using a 0.05 Significance Level for Variable Retention

**Table 4.**
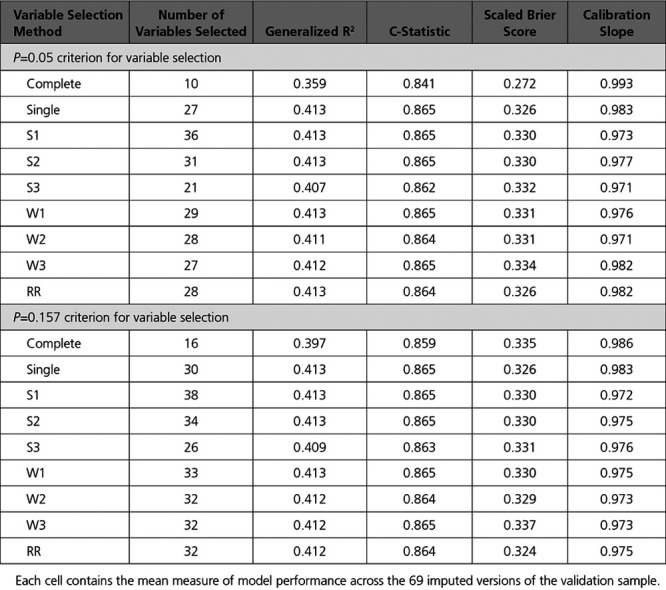
Measures of Model Performance in the Validation Sample

**Figure 1.**
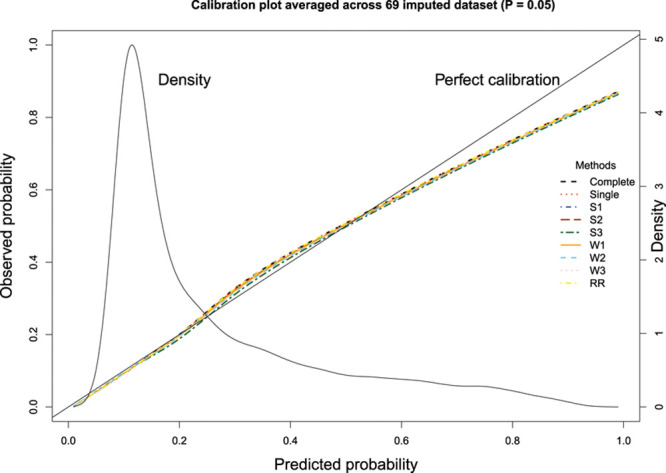
Calibration plot averaged across 69 imputed datasets (*P*=0.05).

The graphical assessment of calibration in the validation sample is described in Figure 1. Deviation of the smoothed calibration plot from the diagonal line with unit slope is indicative of lack of calibration. We have added to this plot a nonparametric estimate of the distribution of the predicted probability of the outcome in the first imputed version of the validation sample using the model selected using Rubin’s Rules (scale on the right vertical axis). All methods of variable selection resulted in models that displayed good calibration when the predicted probability of the outcome was <0.6; however, calibration deteriorated as the predicted probability exceeded 0.6. Calibration was poorest among those subjects with a high predicted probability of mortality. However, as illustrated by the overlaid density plot, there were relatively few subjects with high predicted probabilities of mortality. Differences in calibration between the different models were negligible.

The performance of the different selected models when applied to the validation sample when single mean imputation was used is reported in the top half of Table 5. The generalized R^2^ statistic, the c-statistic, and the scaled Brier score were all lower for the model selected using the complete cases in the derivation sample compared with the models selected using the imputed versions of the derivation sample. In contrast to this, these 3 statistics did not differ meaningfully across the models obtained using different variable selection approaches in the imputed versions of the derivation sample.

**Table 5.**
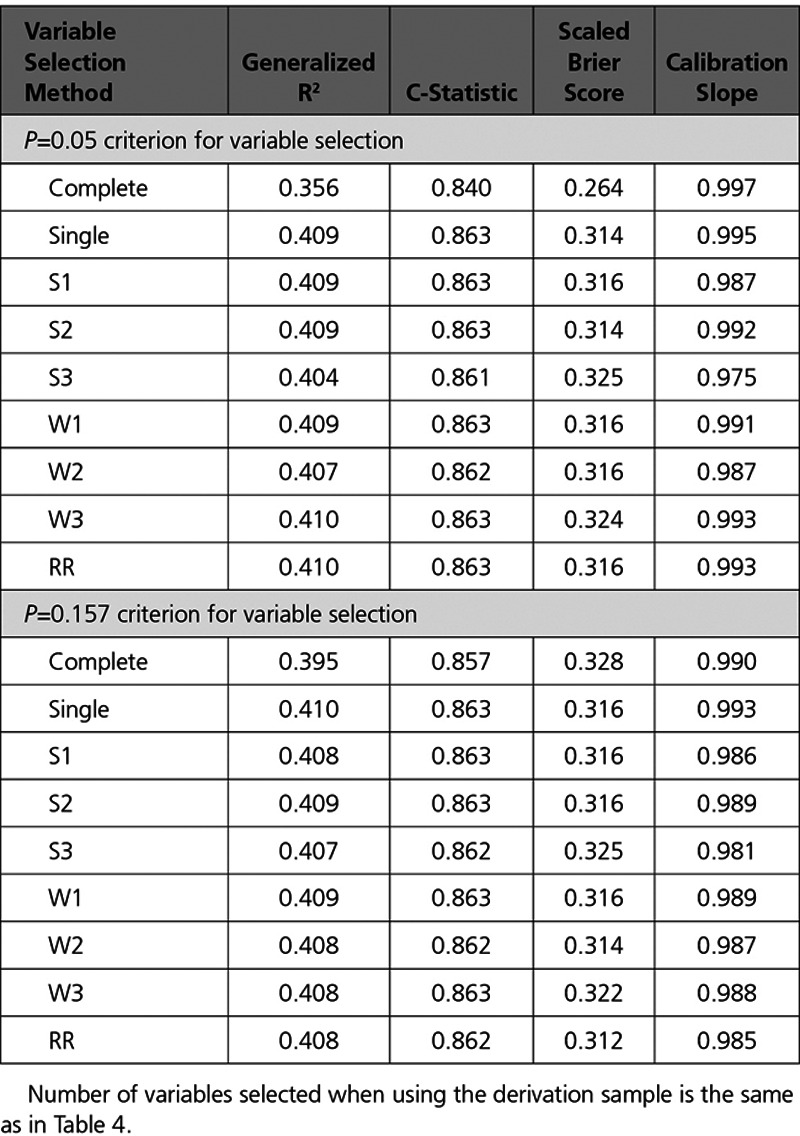
Measures of Model Performance in the Validation Sample With Single Mean Imputation

### Variable Selection Using *P*=0.157 for Variable Retention

When using a 0.157 significance level for variable retention, 3 variables were not selected using any of the variable selection approaches, while 14 variables were selected using all 9 variable selection approaches. The numbers of selected variables for the different variable selection methods were: 16 (complete case selection), 26 (S3), 30 (single sample selection), 32 (W2, W3, and Rubin’s Rules), 33 (W1), 34 (S2), and 38 (S1). The estimated odds ratios and associated 95% CIs for the predictor variables in each model are reported in Table 6.

**Table 6.**
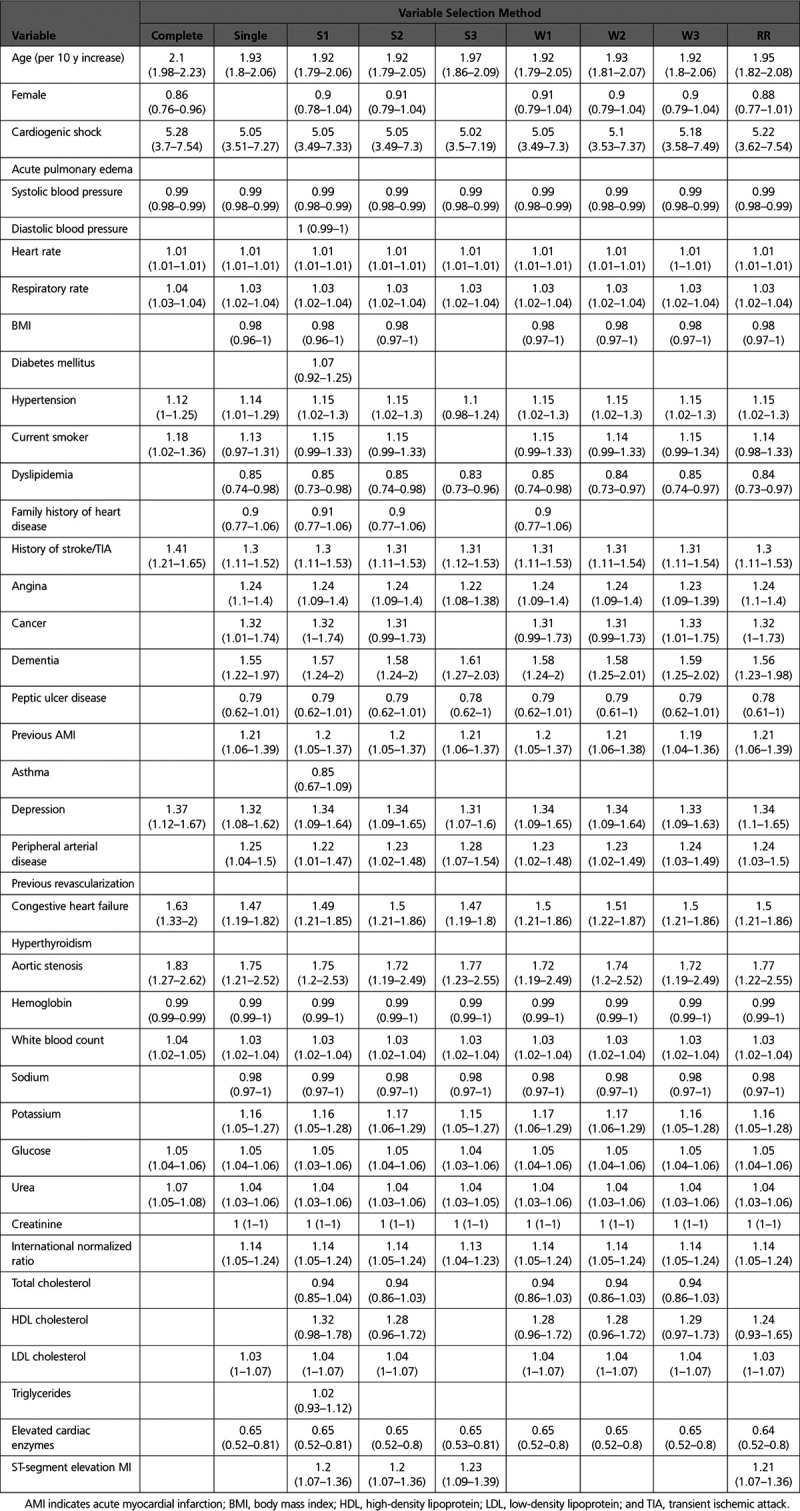
Estimated Odds Ratios and 95% CIs in the Derivation Sample When Using a 0.157 Significance Level for Variable Retention

The performance of the selected models was evaluated in the imputed versions of the validation sample. The mean measures of model performance across the 69 imputed copies of the validation sample are reported in the bottom half of Table 4. Differences in model performance between the model selected using complete cases and the other models was attenuated compared with what was observed when a statistical significance level of 0.05 was used for variable selection.

**Figure 2.**
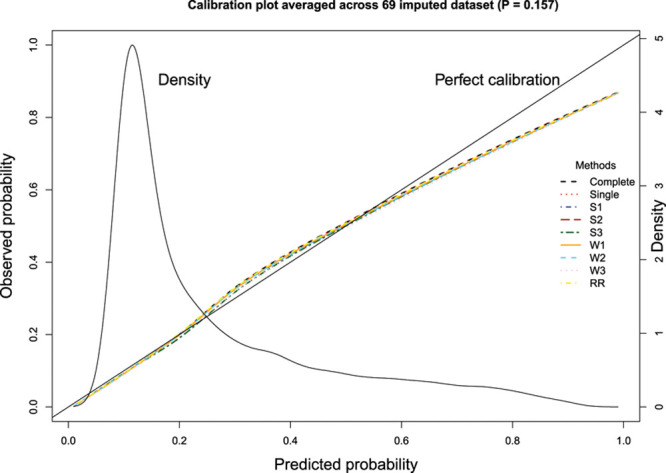
Calibration plot averaged across 69 imputed datasets (*P*=0.157).

The graphical assessment of calibration is described in Figure 2. Results for calibration were similar to those observed when a significance level of 0.05 was used for variable selection.

The performance of the different selected models when applied to the validation sample when single mean imputation was used is reported in the bottom half of Table 5. In contrast to the results obtained when using a significance level of 0.05 for variable selection, the difference between the performance of the model obtained using the complete cases in the derivation sample and that of the models obtained using the imputed versions of the derivation sample were attenuated when a significance level of 0.157 was used for variable selection.

## Discussion

We compared the predictive accuracy of prognostic models developed using different methods for variable selection with imputed data. Model accuracy was evaluated using data from a different temporal era compared with that in which variable selection and model estimation were conducted. The datasets used for model derivation and validation were large and contained a large number of candidate predictor variables. Furthermore, the proportion of subjects with missing data was high. Our primary observation was that the model whose variables were selected using only those subjects with complete data had substantially inferior prognostic ability in the validation sample compared with the models whose variables were selected using the imputed data. The variable selection methods that used all subjects (both those with complete data and those with imputed data) had comparable performance in the validation sample.

There are several reasons why the model selected using the complete cases differed to such a great extent from the models selected using other approaches. First, in the derivation sample, only 13% of subjects had complete data. These subjects may have differed systematically from the entire sample of hospitalized patients. It is plausible that the predictors of mortality differed in this subsample compared with the predictors of mortality in the overall sample. Second, the sample consisting of the complete cases was substantially smaller than the full sample (with a corresponding reduction in the number of observed events). This resulted in a substantially diminished statistical power to identify predictors of the outcome. It was noticeable that the complete case analysis selected substantially fewer predictors than did the other variable selection approaches. The omission of prognostically important variables would result in degraded prediction in the validation sample. These issues highlight the danger of conducting variable selection using only the complete cases.

Variable selection in multiply imputed data has received only modest attention. The most comprehensive study to date is that of Wood et al, who described 9 methods for variable selection.^8^ As noted in the Introduction, they evaluated the variable selection methods in terms of their ability to correctly select variables from the true model and to exclude variables not in the true model. They recommended that variable selection be conducted using a stepwise application of Rubin’s Rules, as this was the only approach that preserved the type I error rate (the probability that a method will incorrectly select a variable that is not part of the true model). We would note that type I error is of less concern when developing a prognostic model. For this reason, we examined the use of a significance level of 0.157 in addition to examining the performance of a significance level of 0.05 for variable retention.

Clark and Altman compared the performance of models for predicting mortality in patients with ovarian cancer.^16^ They found that the model derived using complete cases had worse performance when applied to these subjects than did a model derived using multiple imputation and the full sample when applied to the full sample. We found when using the full validation sample that the models developed using imputed data had superior performance compared with the model developed using the complete cases in the derivation sample. Vergouwe et al compared different methods for developing and validating prognostic models, using prediction of deep venous thrombosis as a test case.^17^ Using the terminology of the current paper, they compared the use of Rubin’s Rules for variable selection with that of S2 and W3. They found that the 3 approaches resulted in similar results for variable selection.

In the current study, we focused on ideal model performance as opposed to pragmatic model performance.^10^ The former refers to the performance of the model in future clinical settings in which all variables are measured and there are no missing data. The latter refers to the performance of the model in future clinical settings in which some subjects have missing data on some variables. Had we been interested in pragmatic model performance, the imputation models in the validation sample would have omitted the outcome variable. Furthermore, we could have developed a set of partial prediction models.^10^ In doing so, we would develop a prediction model for the distinct missing data patterns. Given that our sample consisted of 41 predictor variables, with 643 distinct missing data patterns, such an approach was not feasible in our data.

There are certain limitations to the current study. First, our analyses were based upon empirical analyses in a single dataset. It is possible that different findings would be observed in samples of subjects with different clinical conditions or for different outcomes. However, the datasets used for derivation and validation were large and from temporally distinct periods. This allowed us to assess the temporal transportability of the derived models.^7^ A second limitation, pertaining to the generalizability of our findings, is that the derivation sample was large. We found minimal differences between the variable selection approaches (with the exception of the complete case approach) in terms of their prognostic ability. It is possible that differences between the variable selection approaches would be amplified in small samples. A third limitation pertains to the use of data from an earlier era (1999–2001 and 2004–2005). The distribution of patient characteristics and patterns of care for patients with acute myocardial infarction may differ between this era and the current era. It is conceivable that the selected predictor variables and the rate of missing data would differ in a more recent era. However, the use of these data was to illustrate statistical issues in variable selection when using multiple imputation. The objective of the current study was not to derive clinical prediction models for use in current clinical practice.

We illustrated that, apart from variable selection using the complete cases, the competing variable selection methods produced prognostic models that had comparable performance when evaluated in a temporally distinct validation sample. Despite the similar performance of the different models, we would argue for the use of the method based on the application of Rubin’s Rules for variable selection. Such a selection process results in a final model that has the desirable property that the selected covariates all meet a predefined significance level. This is in contrast to several of the other variable selection methods that resulted in the inclusion of nonsignificant covariates once the final model was estimated using Rubin’s Rules. A limitation to the use of Rubin’s Rules (and to the S1, S2, S3, and W3 methods) is that they require user-written software, and they are typically not available in standard statistical software (though the mim stepwise procedure in the mim package for Stata can be used for stepwise variable selection in multiply imputed data). In contrast to this, the single method and the W1 and W2 methods can be easily implemented using conventional statistical software packages.

## Sources of Funding

This study was supported by ICES, which is funded by an annual grant from the Ontario Ministry of Health and Long-Term Care (MOHLTC). The opinions, results, and conclusions reported in this paper are those of the authors and are independent from the funding sources. No endorsement by ICES or the Ontario MOHLTC is intended or should be inferred. This research was supported by an operating grant from the Canadian Institutes of Health Research (CIHR; MOP 86508). Drs Austin, Ko, and Lee are supported in part by Mid-Career Investigator awards from the Heart and Stroke Foundation. Dr Lee is the Ted Rogers Chair in Heart Function Outcomes. Dr White was supported by the Medical Research Council Unit Programme MC_UU_12023/21. The Enhanced Feedback for Effective Cardiac Treatment data used in the study was funded by a CIHR Team Grant in Cardiovascular Outcomes Research (grant numbers CTP 79847 and CRT43823).

## Disclosures

None.

## Supplementary Material
