# Supplementary material for: Effect of Variable Selection Strategy on the Performance of Prognostic Models When Using Multiple Imputation
Source: Circ Cardiovasc Qual Outcomes. 2019 Nov 13;12(11):e005927. doi: 10.1161/CIRCOUTCOMES.119.005927 (PMC7665277; doi:10.1161/CIRCOUTCOMES.119.005927)
Supplement: Supplementary file 1 [file hcq-12-e005927-s001.pdf]

SUPPLEMENTAL MATERIAL

Appendix. Summary of variable selection methods

Name	Description
Complete	Conduct variable selection in the complete cases – those subjects with no missing data
Single	Conduct variable selection using only one imputed dataset (e.g., the first imputed dataset)
S1	Conduct variable selection in each imputed dataset. Select those variables that were selected in at least one imputed dataset
S2	Conduct variable selection in each imputed dataset. Select those variables that were selected in at least half of the imputed datasets
S1	Conduct variable selection in each imputed dataset. Select those variables that were selected in all of the imputed datasets
W1	Stack the M imputed datasets. Conduct variable selection using weights w = 1/M
W2	Stack the M imputed datasets. Conduct variable selection using weights w = (1-f)/M, where f denotes the proportion of missing data across all variables
W3	Stack the M imputed datasets. Conduct variable selection using weights w_j = (1-f_j)/M, where f_j denotes the proportion of missing data for variable X_j
RR	Conduct variable selection using applications of Rubin's Rules for assessing the statistical significance of variables
